# Knapping force as a function of stone heat treatment

**DOI:** 10.1371/journal.pone.0278643

**Published:** 2022-12-02

**Authors:** Klaus G. Nickel, Patrick Schmidt

**Affiliations:** 1 Department of Geosciences, Applied Mineralogy, Eberhard Karls University of Tübingen, Tübingen, Germany; 2 Department of Geosciences, Prehistory and Quaternary Ecology, Eberhard Karls University of Tübingen, Tübingen, Germany; Semnan University, ISLAMIC REPUBLIC OF IRAN

## Abstract

We propose a quantitative framework for understanding the knapping force requirements imposed by different raw materials in their unheated and heat-treated states. Our model interprets stone tool knapping as being the result of cracks formed during the first impact with a hammer stone, followed by continued stressing of these cracks that eventually leads to flake detachment. We combine bending strength, indentation fracture resistance and “Griffith” crack lengths of flint and silcrete to obtain functions identifying critical forces for flaking without or after heat treatment. We argue that these forces are a key factor for understanding the “knappability” of different raw materials, because only forces with 100N or less can be used for very precise strike control. Our model explains for the first time why experimental knappers frequently observe that flint (a stronger material, which, in our case, has a strength above 100 MPa) is easier to knap than silcretes (which is relatively weaker with strength values at or below 60 MPa). Our findings allow for understanding the differences between heat-treated and untreated flint and silcrete in terms of knapping quality, and they allow to compare the qualities of different raw materials.

## Introduction

In archaeology, the study of materials used for making stone tools (commonly called stone knapping or tool knapping) provides important insights into the organisation and behaviours of ancient peoples (e.g. [1–3]). It is commonly accepted that different raw materials (i.e. rocks used for stone tool making) have different properties and present different constraints for knappers (see for example [4]). One such constraint is commonly called “knapping quality”. To explain the knapping quality of different raw materials, experimental knappers often cite subjective parameters such as the ease of flaking and the predictability of fracturing.

Isotropic brittle materials (showing no or only weak crystallographic cleavage and no microstructural layering) are relatively easy to handle by knappiers, as they tend to fracture conchoidally. Stone tool making using such materials relies on the knapper’s intuitive understanding of how such conchoidal fractures behave.

Unfortunately, it is still poorly understood how knapping quality can be measured objectively. Several studies investigated the relations of fracture propagation with the geometry of the fractured materials [5], the angle and magnitude of applied force [6–9] or the type of material transmitting the force [10]. These studies, all performed on standardized glass cores, not on rocks, convincingly demonstrated the importance of detachment geometry for stone knapping. Mechanical properties and their meaning for knapping quality have not been investigated. Here, we propose a framework for understanding one factor that contributes to knapping quality: the knapping force requirements imposed by different raw materials.

For this, we use data obtained on heat-treated silica rocks flint and silcrete. Both rocks were used in several parts of the world. Many archaeological sites in Europe, America, Asia and Australia document heat treatment of these rocks (for an overview, see: [11]). Studying unheated silica rocks and the changes they undergo when heated, allows to understand the effects of changing mechanical properties while reducing other variables like inter-sample heterogeneity.

One of the mechanical properties of interest is the stress, at which a material fails by fracturing, called strength. In an ideal glass, a theoretical strength value can be calculated from atomic binding energies and might be expected to be an important parameter for evaluating knapping quality. However, rocks are complex materials. They contain flaws such as macro- and intergranular pores [12–14]; crystal dislocations [15, 16]; inclusions [17], grain or domain boundaries, so that internal structural features are decisive for the strength of those apparently conchoidal fracturing raw materials. The inadequacy of strength for explaining knapping quality is illustrated by an apparent contradiction that is commonly noticed by experimental knappers working with rocks flint and silcrete: flint is subjectively described as being the better raw material for knapping [18] although its strength is higher than that of silcrete by a factor of three [19].

Another mechanical property is fracture toughness—the resistance of a material against the elongation of an existing flaw or crack. Together with the statistics of flaws in a material (number, distribution, shape, length,…), fracture toughness has an influence on fracture strength (see for example: [20]) and for this reason may be a key factor for understanding knapping quality.

In this study, we therefore use indentation resistance measurements together with basic elastic properties to calculate a proxy for the true fracture toughness, because the direct experimental determination of it is very elaborate (e.g. [21]). We use these indentation resistance measurements to calculate the stresses necessary for fracturing and, by this, to explain how much force is needed for the knapping of different stones. This quantitative understanding, derived from unheated and heat-treated rocks, also has important implications for our understanding of the differences between other raw materials.

## Theoretical background

The calculations we present here are not meant to simulate the exact processes taking place during knapping. Knapping of rocks for stone tool making is a process based on impact and shear (see for example [22]). Testing for shear strength requires an instrumental setup that is not available to us.

The framework we propose for comparing the fracture mechanisms of different rocks is based on tension. This is reasonable, because the dynamics we identify are sufficiently similar so those occurring in shear. The validity of the analogy may already be inferred from the definitions of tension *σ* and shear stresses *τ*,


σ=E⋅ε (Eq 1)

and


τ=G⋅γ (Eq 2)

in which *E* and *G* are the elastic moduli (see for example [23]), acting as linear proportionality factors of the stresses with the tensional strain *ε* and the shear strain *γ*. In tension, strain is the relative value of a one-dimensional length change (Δl/l_0_), while for shear, it is an angle of deformation change. Only the measurement of the former is straightforward, which is why we use it here. In terms of a material’s elastic behaviour, tension and shear moduli are related by (see for example [23]):

$$G=\frac{E}{2(1+\mu )}$$ (Eq 3)

Tensional and shear moduli in Eq (3) are related via the Poisson ratio *μ*, describing the relative thickness change for a given length change. The value lies in the range of 0.2 to 0.3 for most brittle materials (i.e. *G* ≈ 0.4 ∙ *E*). Thus, the following rationale on tension is also fit to make statements on shear.

Since we use tensional strain for our approximations, tensional stress *σ* and K_Ic_, defined as the resistance against crack prolongation in uniaxial tension, are the appropriate parameters for our evaluation and also the parameters usually reported by the stone tool community [24, 25]). When discussing *K*_*Ic*_ as a factor for knapping quality, the processes to be taken into account should therefore rely on tensional stress. *K*_*Ic*_ is traditionally determined through relatively expensive single-edge notch-beam (SENB), or related, tests [26]. A widely used, much more cost-effective test for a crack resistance parameter is via indentation with a sharp indenter (typically Vickers-diamonds), creating cracks extending from the edges of the indents. Even though the exact nature of the cracks obtained is in dispute (see below), Lawn’s [27] analysis of the indentation crack length *c* as a function of the resistance against crack elongation *K*_*Ic*_ and as a function of the loading force F is still valid:

$$C^{\frac{3}{2}}=\frac{\chi \cdot F}{K_{I_{C}}}$$ (Eq 4)

This formulation is found–with differing indices of *K*–in a number of fracture mechanical papers investigating such cracks (e.g. [28, 29]). The problem with the formulation comes from the function *χ* because the stress field beneath an indentation is a complex phenomenon related to Boussinesq (point-contact) and Hertz (ball contact) problems: it is not constant throughout a volume of indented material and involves the build-up of residual stresses. Thus, *K*_*Ic*_ measured through indentation is not identical with *K*_*Ic*_ measured through SENB-tests. Indentation *K*_*Ic*_ values are therefore not accepted by many authors as an accurate test method (for an extended discussion see [30]). Indentation testing for *K*_*Ic*_, should better be named *indentation fracture resistance* [21], but will allow comparisons between materials and show trends that reflect real fracture toughness. There are new approaches to understand the energetics and meaning of indentation cracks. However, they are not developed enough yet to establish reliable data for our study [31–33]. We therefore apply the widely-used formulation of Niihara et al. [34]

$${\text{K}}_{\text{Ic}}=0.067\text{H}\sqrt{\text{a}}{\left(\frac{\text{E}}{\text{H}}\right)}^{0.4}{\left(\frac{\text{c}}{\text{a}}\right)}^{-\frac{3}{2}}$$ (Eq 5)

to quantify a material’s toughness and call it—as proposed by this author—*K*_*Ic*_ (with *H* = Vickers hardness, *a* = the diagonal of the indent in the material).

A third assumption we make is that *K*_*Ic*_ can be interpreted in the light of Weibull’s [35] statistical interpretation of fracture. Weibull proposed a way to understand and handle mathematically the distribution of different strength values of a material, because in real-world situations, tests of brittle materials show a variation of strength values. Standard tests for rocks or ceramics commonly utilize standard-sized bending bars tested in 4-point bending [36, 37]. Weibull theory requires a large number of tests to make well defined statements on statistics of the distribution of stress values. Such statistical reasoning normally requires >25 samples, although a smaller number is acceptable. Smaller sample sizes effectively increase the uncertainty of the parameters derived from Weibull plots (cf. Fig 10 of [38]). Our data sets (cf. Schmidt et al. [19], table 3) typically contain ten samples. From such a distribution, the Weibull modulus *m* is calculated as a quantitative measure of the distribution. Low values of *m* (< 5) are associated with a wide distribution: there is a high variability in the stress at which a piece of this material fail. Low *m*-values correspond to large error bars from standard deviations in classic evaluations of mean failure stresses from lists of individual experimental results. High values of *m* (> 10) indicate a fairly reliable prediction of the failure stress (“small failure stress error bars").

The Weibull modulus *m* reflects the distribution and size of faults pre-existing in the material, which cause failure at a certain stress. These faults may be cracks, pores, inclusions, etc. Typical Weibull plots show the probability of failure over material strength and allow the extraction of *m*-values and an estimation of a *σ*-value with a standardized failure probability of 63.2%, the so-called characteristic strength *σ*_*0*_. The latter can be used to compare different materials with each other. For comprehensive and more recent overviews of Weibull theory and how to extract these values, the reader is referred to papers of Danzer and colleagues (e.g. [38–40]). For those preferring text books we recommend [26].

To transfer the obtained characteristic strength from the tested suite with one particular effective volume to samples of interest with a different volume, we use the standard Weibull relation [41]

$$\frac{\sigma_{1}}{\sigma_{0}}={\left(\frac{V_{0}}{V_{1}}\right)}^{\frac{1}{m}}$$ (Eq 6)

Here, *σ*_*0*_ and *σ*_*1*_ are related through their effective tested volumes *V*_*0*_ and *V*_*1*_ and the material’s Weibull modulus *m*. The equation reflects the statistical fact that a larger volume is expected to contain a larger amount of faults and a higher probability to contain a larger fault. Brittle materials normally fail at the largest fault of the stressed volume. Therefore, samples with larger volumes fail at lower stresses. A detailed discussion of the effective volume of samples in bending tests has been given in [41, 42]. For our set-up, the effective volume in 4-point bending *V*_*eff*_^*4-PB*^ is accordingly taken as

$$V_{eff}^{4-PB}=\frac{m+2}{4{\left(m+1\right)}^{2}}\cdot S_{1}\cdot b\cdot h$$ (Eq 7)

with *S*_*1*_ = span of lower bearings in 4-point bending, *b* = sample width, *h* = sample height (see for example [41]).

Our discussion of the potential force needed for knapping of different materials requires tests to be performed for a given standardized sample geometry. Any extrapolation to other sample volumes is therefore performed using Eqs (6) and (7). This is a necessary procedure for many rock test suites, because coarse-grained materials ask for larger test samples. This is so because it is more difficult to manufacture samples from coarse-grained rocks, and coarser rocks also require larger volumes for the samples to be representative of the whole rock (e.g. in coarse Silcretes). Finer-grained rocks allow producing samples with smaller volumes (e.g. flint nodules). The comparison of the volume-dependent quality strength for such different sets of sample has therefore to be done by normalization of the results to a standard volume.

## Materials

Here, we use *K*_*Ic*_ data from Schmidt et al. [19] for our argument on knapping force. These data were produced in the framework of a study on silica rocks flint and silcrete, comparing their mechanical properties when unheated and heat-treated to different temperatures. Missing information not reported in the publication, e.g. standard deviations, are provided in Table 1. In the present study we only used those data from Schmidt et al. [19] for which the full set of *K*_*Ic*_ and *σ*_*1*_ values was obtained. In tables 3 and 4 of Schmidt et al. [19], the authors reported only *σ*_*c*_ data and Weibull modulus *m* for samples with different volumes. For this study, we normalized these volumes to a common test volume of 30 mm^3^, as described above using Eqs (6) and (7). With those newly derived data, we extracted *σ*_*0*_ from the respective Weibull plots.

**Table 1:** Selected data from Schmidt et al. [19] used for this study.

Sample N^o^	Rock	Indentation force (N)	E (GPa)	Mean of σ_1_ (MPa)	σ_0_ (MPa) for V_eff_ = 30 mm^3^	Weibull Modulus m (corr. coeff, r)*	Mean Crack length c (μm)	K1c (MPa* √m)
VC-12-05	Flint unheated	200	74.82 ± 0.9	166.5 ± 21.7	189	2.61 (0.8688)	382 ± 11.5	1.71 ± 0.08
VC-12-05	Flint unheated	100	74.82 ±0.9	166.5 ± 21.7	189	2.61 (0.8688)	240 ± 8.1	1.77 ± 0.09
VC-12-05	Flint unheated	50	74.82 ±0.9	166.5 ± 21.7	189	2.61 (0.8688)	145 ± 5.4	1.85 ± 0.1
VC-12-05	Flint 250C	50	75.93 ± 0.4	154.3 ± 21.2	173	2.82 (0.9208)	144 ± 3.2	1.86 ± 0.06
VC-12-05	Flint 250C	100	75.93 ±0.4	154.4 ± 21.2	173	2.82 (0.9208)	291 ± 5.9	1.32 ± 0.04
VC-12-05	Flint 250C	200	75.93 ±0.4	154.3 ± 21.2	173	2.82(0.9208)	402 ± 4.5	1.59 ± 0.03
VC-12-05	Flint 300C	100	76.35 ± 0.8	154.8 ± 16.6	146	5.74 (0.974)	288 ± 11.1	1.36 ± 0.08
VC-12-05	Flint 350C	100	76.36 ± 0.5	133.7 ± 9.2	125	7.74 (0.9805)	383 ± 16.9	0.89 ± 0.06
WK-13-08	Silcrete unheated	100	67.42 ± 3.4	52.7 ± 5.3	57	5.02 (0.9421)	232 ± 12.3	1.85 ± 0.14
WK-13-08	Silcrete 250C	100	64.4 ± 0.5	50.8 ± 3.3	54	5.28 (0.9208)	251 ± 17.9	1.67 ± 0.21
WK-13-08	Silcrete 350C	100	63.72 ± 13.2	58.1 ± 7.6	70	3.72 (0.9724)	287 ± 8.9	1.31 ± 0.06
WK-13-08	Silcrete 450C	100	63.83 ± 4.3	60.5 ± 4.7	59	6.96 (0.9908)	303 ± 7.6	1.20 ± 0.06
WK-13-08	Silcrete 600C	100	58.77 ± 4.2	53.0 ± 5.3	57	5.7 (0.9845)	285 ± 18.1	1.29 ± 0.11
WK-13-13	Silcrete unheated	100	76.43 ± 0.9	43.6 ± 6.8	73	2.86 (0.9671)	255 ± 16.4	1.71 ± 0.19
WK-13-13	Silcrete 250C	100	75.18 ± 2	41.2 ± 5.2	48	6.75 (0.9536)	271 ± 12.8	1.54 ± 0.12
WK-13-13	Silcrete 350C	100	68.13 ± 2.4	36.3 ± 2.5	42	8.32 (0.9724)	280 ± 15.8	1.38 ± 0.12
WK-13-13	Silcrete 450C	100	59.92 ± 2.9	34.3 ± 2	36	9,18 (0.9908)	276 ± 12.3	1.33 ± 0.09
WK-13-13	Silcrete 600C	100	49.88 ± 3.3	32.1 ± 1.8	34	9.57 (0.9845)	271 ± 12.8	1.22 ± 0.09

Uncertainties of the reported values are standard deviations calculated from all measurements made at these temperature steps (as reported in [19]). As σ_0_ values are recalculated from σ_1_, the deviation values of the latter are also the best indication for the precision of the former.

* Weibull-coefficient *m* is taken from Weibull plots; its correlation coefficient *r* is given in brackets.

In addition, we conducted new indentations on five of Schmidt et al.’s [19] flint samples (VC-12-05), unheated and heated to 250°C and 400°, to obtain *K*_*Ic*_). These measurements were made on their samples and conducted with the same experimental setup but different indentation forces (50 N and 200 N instead of the 100 N in [19]). The data we use here are summarized in Table 1.

## Results and discussion

**Fig 1:**
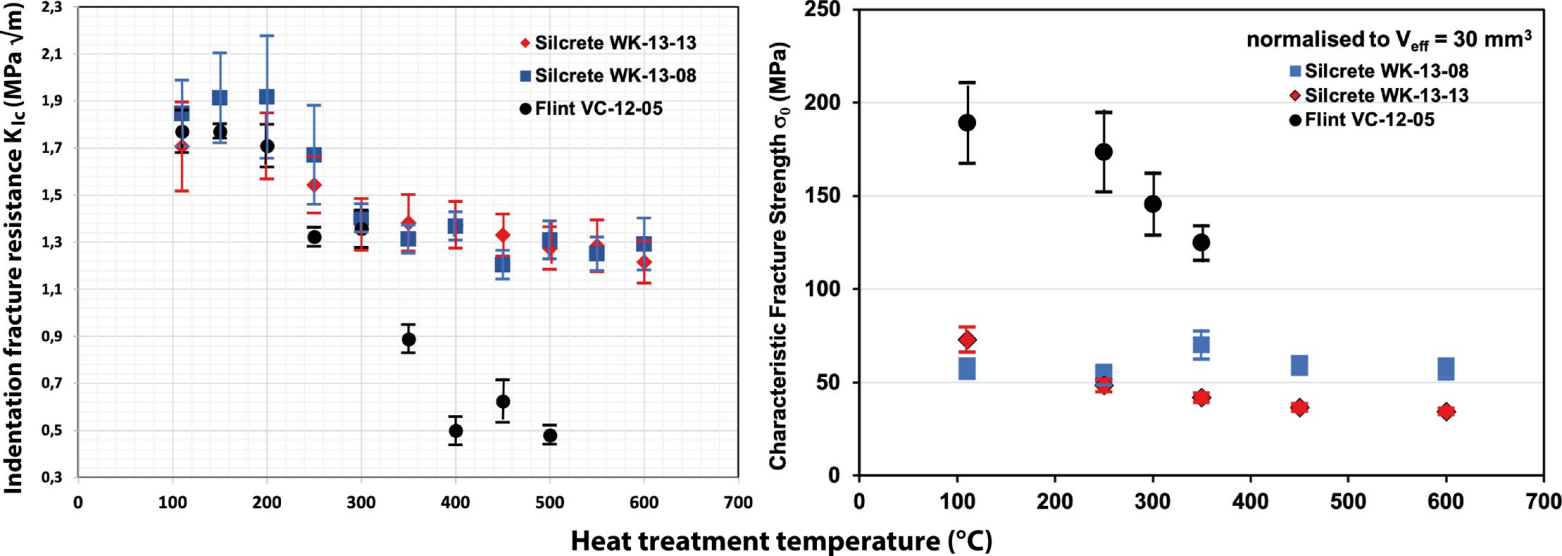
Plot of indentation fracture resistance *K*_*Ic*_ (a) and characteristic strength *σ*_*0*_ (b) values of Table 1 as a function of the temperature of treatment. Note that *K*_*Ic*_ values in (a) are also reported for those temperature steps for which no *σ*_*0*_ is available. The error bars in (b) are taken directly from the corresponding data tables of Schmidt et al. [19], from which *σ*_0_ is calculated.

Fig 1 shows two plots of the evolution of indentation fracture resistance *K*_*Ic*_ and characteristic strength *σ*_*0*_ with increasing heat treatment temperatures, generated from the data in Table 1. There is a trend of decreasing *K*_*Ic*_ upon heat treatment in both rock types (Fig 1A). Thus, heat treatment leads to a reduction of the force needed to elongate pre-existing cracks in flint and silcrete. In contrast, Fig 1B indicates moderate changes of *σ*_*0*_ in flint and weak or no changes in silcrete. At first glance, this seems to be in contradiction with empirical observations made on flint and silcrete. Flint is commonly known as a material that requires less knapping force than silcrete. Since *σ*_*0*_ is mathematically the stress needed to break the samples (stress = force/area), the higher *σ*_*0*_ is often thought to have to reflect the actual force requirement during knapping.

*K*_*Ic*_ in Fig 1A is calculated from crack length *c* using Eq (5). The *c* values reported in Table 1 were obtained by a sharp point contact after indentation with 100 N force and unloading. The formation of cracks due to indentations is observed for both blunt and sharp indenters [29, 43, 44], median and Palmquist cracks are typical for sharp indentations, cone cracks are typical for blunt (ball) indentations. Similar cracks are also observed for impacts with indenters (used for material testing) with these shapes [45]. The influence of geometrical constraints, such as the proximity to edges or the angle of the blow, can further complicate crack formation and crack direction (for example in edge chipping [46]). However, there is still a strong connection between indentation-produced features and knapping: In indentation, the force used is loaded and then unloaded. The so formed cracks are either stable cracks formed during loading or unloading features due to residual stresses created in the material. With respect to knapping, Tsirk [47] notes: “Since a flake detachment may require less than a millisecond, it seems to make no sense to talk about follow-through forces in knapping. Yet experienced knappers will not question the significance of a follow-through in knapping”. Tsirk’s observation may be best understood in the light of the following mechanical sequence: a crack is developing during the knapping blow or, alternatively, the material already contains a crack created by a previous blow (in case of a succession of blows) that is enlarged by the knapping blow. This crack then remains under stress opening conditions because the hammer is still exerting force. In this way, the initially formed crack acts as an origin of the final flaking event (the flake detachment).

Knapping is an activity relying on many variables, such as the shape and mechanical properties of hammer stones and cores, angle of blow and others [48]. However, modelling of the energetics involved in knapping my follow the argumentation of Kerkhof and Müller-Beck [49], who considered the energy needed to extend a pre-existing crack into a cone crack. Following this argumentation, knapping force might be explained by estimating the surface energy and/or strain energy release rates of the material. Similar approaches may also be developed studying Lawn´s recent review [50].

It is known from the treatment of Bussinesq- and Hertz-Problems [29] that the tension in the material under a pressure point is responsible for the appearance of indentation cracks. Details of the current state of the art of research on indentation cracks were reviewed by Cook and Pharr [51]. In this paper, we only aim to explain how the force of a flake-removing blow is influenced by such indentation cracks. We use this as proxy for “knappability” and/or the ease of flaking.

To do so, we will treat the problem with the simplest approximation: using the *K*-value in Eq (4) strictly as *K*_*Ic*_ and the crack formed during indentation as one under simple tension (sticking to the definition of *K*_*Ic*_, i.e. simple uniaxial tension perpendicular to the crack extension). Thus, we can use the basic relation between strength *σ*_*c*_ and fracture toughness *K*_*Ic*_ in ideally brittle materials under tension in the form of one type of the Griffiths relation [52]:

$$K_{Ic}=\sigma_{c}\cdot Y\cdot \sqrt{a_{c}}$$ (Eq 8)

Here, *Y* is a factor adjusting for geometrical differing faults and *a* is the crack length. It should be noted that the definition of a geometric correction term may be different in different references. Lawn [27] names *Y* in its Greek form (ψ) to include a π-term, which arises from the analysis of the stress intensity factor *K* for a straight crack in an infinite plate, while Danzer [40] keeps this π in the root of the crack length, so that *Y* becomes an additional parameter (i.e. $K_{Ic}=\sigma_{c}\cdot Y\cdot \sqrt{\pi \cdot a_{c}}$). Summaries for standard treatments of crack geometries are given in text books like [26], a more recent discussion is published in [53]. The cracks formed by sharp indenters may be approximated to have the shape of half-penny cracks, which is at least a good approximation to many indentation cracks [54]. This would require a different *Y*-term. However, *Y* just shifts the values in Eq 8 typically by a factor less than 2 and so, for simplicity, we hold the simple term *Y* = π^0.5^ from the Griffith model for straight cracks in infinite plates as sufficient for a basic discussion about ranking material behaviours.

The index *c* in Eq 8 stands for “critical”. Thus, a brittle material that experiences continued stress of the level of *σ*_*c*_ will fail by catastrophic crack elongation, if its fracture toughness has the value of *K*_*Ic*_ and a failure of size *a*_*c*_ is present. The term *a*_*c*_ is then called the Griffith length and represents the size of a crack at which its further propagation is driven by the potential energy resulting from the crack growth, which is greater than the increase in surface energy needed for further crack opening (in other words, at *a*_*c*_ the crack runs all through the material). *K*_*Ic*_ is a material property and therefore independent of volume, but the σ_*0*_ values reported in Table 1 are representative for an effective tested volume *V*_*eff*_ of 30 mm^3^. It may easily be extrapolated to other volumes using Eq 6.

An effective tested volume of only 30 mm^3^ may seem small at first glance. However, 30 mm^3^ is a typical volume for standard beams of brittle inorganic materials tested by 4-point bending. The effective tested volume here is much smaller than the volume of the total beam, because there is a distribution between tension on one side and compression on the opposite face of the beam. The statistics of failure distribution are such to correspond to a crack-opening condition within the zone of tensional stresses as described by the Weibull module *m*, which leads to Eq (7), effectively limiting the tested volume. This is true for every bending test and, in knapping, the follow-through movement is expected to create bending stresses just as limited to a small zone. We therefore consider our 30 mm^3^ volume to lie within the range of realistic volumes to interpret a real-world knapping situation.

From the discussion above, it should be clear that we do not make predictions that can be used *sensu stricto* for knapping of a certain rock type. All used approximations mentioned above (pure tension instead of shear, indentation fracture resistance instead of exact *K*_*Ic*_ values, neglecting crack shape and residual stresses, restriction to one effective volume, etc.) contribute to the difference of our strength values as compared to strength values relevant to real world knapping. However, our values can be used to compare different raw materials.

For the purpose of the current study, this is acceptable because it is generally impossible to make statements such as “flint has a strength of X MPa”. This is only possible for one individual piece of rock under a certain stress or, using Weibull statistics, for a strength of particular statistical variation (e.g. “mean” or “characteristic” strength) from a set of data. Nonetheless, we show in our modelling the influence of changes in *K*_*Ic*_, which were imposed by heat treatment, on the flaking conditions and eventually on the forces necessary for it. The direction of effects and the orders of changes can be expected to be realistic and in this way allow statements on the “knapping quality” of lithic materials.

**Fig 2:**
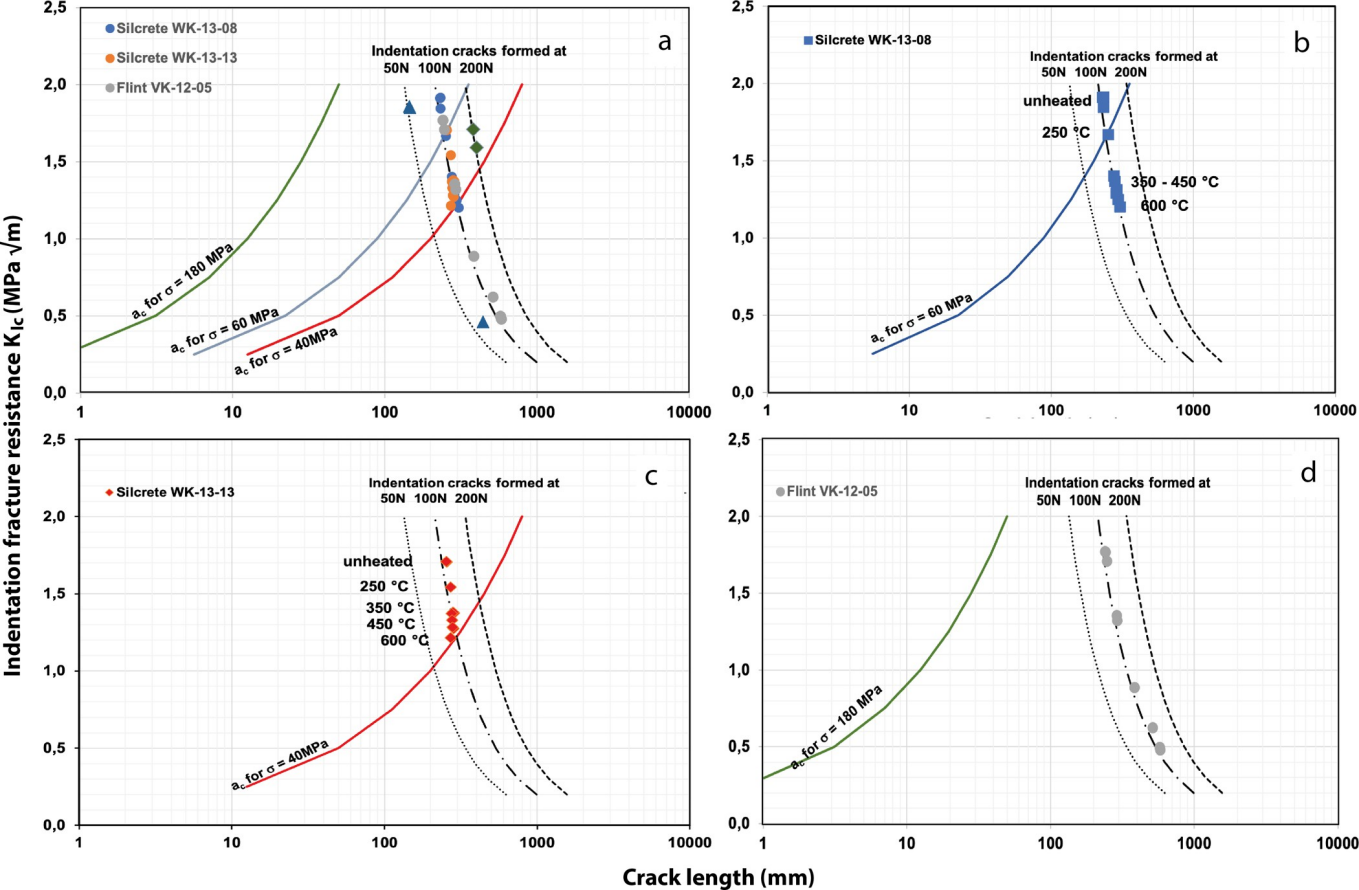
Diagrams reporting crack length as a function of indentation fracture resistance. Two sets of lines are displayed: critical flaw sizes for a given uniaxial tension stress from Griffith Eq (8) and fits of indentation crack length from Eq (4) for a given indentation force. A more detailed explanation can be found in the main text.

The Griffith relation (Eq 8) is thus used to understand knapping conditions. This can be visually appreciated in Fig 2. Using the plausible strength values from our experimental data (164 MPa for flint, the range of 40 to 60 MPa for silcrete), Eq 8 gives critical “Griffith” crack lengths (*a*_*c*_-values) for any assumed or determined *K*_*Ic*_ in the range of values calculated from the indentation experiments. Those are shown as the colored lines in Fig 2A indicating the crack length (*a*_*c*_), which is critical for a given strength, i.e. the condition for flaking as a function of the *K*_*Ic*_.

It can be seen that this is different for materials with differing strengths. The differing material strengths (flint at 164 MPa and silcrete variation between 60 to 40 MPa) are displayed in Fig 2A by different lines. Note that stronger materials are associated with smaller a_c_-values.

In Fig 2A, there is another set of (broken) lines, which are the result of a fit following Eq (4) to the data (dots in Fig 2A) from the indentation experiments (Table 1). The form of the fit matches well with the 100 N-data, validating the use of Eq (4). Therefore, we infer that all of our materials–made from almost pure silica–have an identical value of *χ*. With this fixed value, we can use Eq (4)) to extrapolate to forces other than the 100 N used in other experiments of Table 1. The blue triangles in Fig 2A from flint indentation experiments at 50 N and 200 N agree well with this assumption (grey broken lines in Fig 2A).

Fig 2B–2D shows, for each of our samples separately (two silcretes and one flint), the crack formation by indentation forces *F* and the critical crack size *a*_*c*_ as calculated from the Griffith equation. These graphs predict the conditions at which cracks become critical: the conditions of complete failure (flaking) lie directly on and to the right of the continuous colored curves. If the grey broken line for a given indentation force intersects the Griffith-line, a sufficiently low *K*_*Ic*_ necessary for flaking is reached.

As show in Fig 1A, lowering of *K*_*Ic*_ is a function of heat treatment. Accordingly, specific heat treatment temperatures are indexes in Fig 2B and 2C. Looking first at silcrete (Fig 2B and 2C), we observe intersections of the central grey broken line with the Griffith-line for their strength in 100 N-experiments. Heat treatment temperatures for those intersections are different for the stronger and the weaker Silcrete (WK-13-08: 60 MPa and WK13-13: 40 Mpa). The Silcrete with 60 Mpa strength intersects at about 250°C of treatment, while the weaker silcrete intersects only at about 600°C.

If the blow force used were fixed to 10 kg-force (the 100 N line in the diagrams), those heat treatment temperatures would be necessary to allow flake detachment. Alternatively, one may follow the paralleling grey lines for different forces. Here, the 200-N-line in Fig 2B already lies to the right of the Griffith-line for an untreated condition. This means that a blow exceeding ~15 kg-force (150 N) would be sufficient to flake the untreated material. The left grey line (50 N) stands for a weaker 5 kg-force blow. Such a blow would be successful in flake removal only after heat treatment at about 350°C.

The weaker Silcrete (Fig 2C) intersects the 100 N-line only for very high temperatures of heat treatment (600°C). Therefore, a material heat-treated at lower temperatures would require a higher blow force (exceeding 20 kg-force) to knap it. To get an impression of the meaning of those force values, it is possible to consider forensic studies of fist punches [55]. An Olympic boxer may exert a maximum force of more than 5000 N, but untrained hitters only reach 300 to 800 N. These are maximum values of poorly controlled hits, not well controllable blows that must be repeated several times (like a knapper has to execute blows). Thus, the 100 N-line may well be a realistic choice for an indication of knapping forces.

In Fig 2D, the flint sample has a Griffith line way below the grey lines. Thus, flint can be flaked with indentations of low forces, heat-treated to lower its *K*_*Ic*_ or not. Applied to stone knapping, this means that it is easy for a knapper, i.e. not much force is needed. Heat treatment may improve this to the stage, at which it even becomes delicate to knap the material. This, of course, depends on the strength of the individual flint resource used. There are cases, where flint bending strengths of below 100 Mpa were reported [56]. For such materials, heat treatment, event at higher temperatures, may be beneficial.

## Conclusions

We introduce a model that aims at providing an understanding of the knapping force requirements imposed by different stone raw materials. This model is not meant to predict exact knapping forces required during stone knapping, which are also dependant on volume, geometry and gesture. Our study highlights the changes induced by heat treatment at different temperatures and their effect on different materials (flint, e.g. [57, 58], and silcrete, e.g. [59, 60]). Our model has implications for future studies on raw materials quality because:

- It is the first physical model to directly address the force at which knapping may lead to flake detachment, a quality we infer to be a key parameter for all discussions of the terms “knappability” or “knapping quality”. We argue that required knapping force is a key factor for past humans’ preference of one material over another or for their decision to heat-treat these materials to achieve better knappability.
- In all our silica rocks, we found that shorter indentation cracks are formed when lower forces are applied, which is intuitive. However, the length of indentation cracks is not a function of the strength of the material, a finding that is highly counter-intuitive (silcrete with a strength of 57–73 MPa allows cracks of 225–232 μm to be formed at 100 N, while flint with a strength of 189 MPa allows similarly long cracks of 240 μm to be formed). Crack length is rather a function of the outcome of Eq (4) and therefore dependant on *K*_*IC*_ which is, before heat treatment, equal or similar for all our tested silica rocks.
- However, different rock strengths cause different critical “Griffith” crack lengths in those rocks (the details are shown in Fig 2). Stronger materials require shorter “Griffith” cracks that lead to failure / flaking.
- This explains for the first time why flint, having greater strength than silcrete, requires less force to be flaked than silcrete, which is a counter-intuitive fact well known by knappers.
- It also explains why different types of silcrete require different amounts of force to be knapped and why the force needed to knap them can be “made” similar by heat-treating them to different temperatures.
- We thus expect that our model makes predictions for other materials that will not be refuted by observations made on experimental knapping.
